# Draft genomes of two *Roseibium* spp. isolated from the coral *Pachyseris speciosa* from a Singaporean reef

**DOI:** 10.1128/mra.00765-24

**Published:** 2024-11-27

**Authors:** Pei Yi Peggy Tang, Aaron An Rong Loh, Dalong Hu, Lindsey Kane Deignan, Stephen Summers, Joao Paulo Andre Pereyra, Rebecca J. Case

**Affiliations:** 1 Singapore Center for Environmental Life Sciences Engineering, Nanyang Technological University, Singapore, Singapore; 2 School of Biological Sciences, Nanyang Technological University, Singapore, Singapore; 3 Singapore Center for Environmental Life Sciences Engineering, National University of Singapore, Singapore, Singapore; 4 Saw Swee Hock School of Public Health, National University of Singapore, Singapore, Singapore; 5 St John’s Island National Marine Laboratory c/o Tropical Marine Science Institute, National University of Singapore, Singapore, Singapore; University of Southern California, Los Angeles, California, USA

**Keywords:** coral, marine microbiology, *Roseibium*, *Pachyseris speciosa*, Singapore, equatorial reef

## Abstract

Two *Roseibium* spp. strains were isolated from skeletal macerates of the Singaporean coral *Pachyseris speciosa* at an ambient high temperature. We sequenced the genomes of SCP14 (JBDZYH000000000) and SCP15 (JBDZYI000000000), which revealed genomes containing genetic elements that play a role in coral health during thermal stress.

## ANNOUNCEMENT

The genus *Roseibium* (previously merged with the genus *Labrenzia*) (1) belongs to the family *Stappiaceae* (class *Alphaproteobacteria*) (2, 3). Members of this genus are associated with Symbiodiniaceae (4, 5), the photosynthetic coral symbionts commonly called Zooxanthellae. Coral-associated *Roseibium* with nitrogen and sulfur cycling, thought to be beneficial to coral symbiosis health, have been proposed as candidate coral probiotics (4, 6). Such biogeochemical properties make them excellent coral probiotic candidates to enhance reef resilience to various environmental stressors.

*Pachyseris speciosa* samples were collected from the seawall at the northern side of Kusu Island, Singapore, and maintained in an outdoor aquaria tank with constant seawater flow. Bacterial isolation was performed as described by reference (7), with both isolates incubated at 33°C. Individual bacterial colonies were subcultured to ensure purity, and a single colony was used to inoculate marine broth (Difco 2216) for DNA isolation and sequencing.

Genomic DNA was extracted with the DNeasy PowerSoil Pro Kit (Qiagen, Hilden, Germany). DNA library preparation used the Accel-NGS 2S Plus Low Input DNA Library Prep Kit (Swift Biosciences, Ann Arbor, USA). Sequencing was performed using an Illumina HiSeq X Ten platform, version 2.5 (Illumina, San Diego, CA, USA), generating 150 bp paired-end reads. DNA library preparation and sequencing were performed by the SCELSE Sequencing Facility, NTU.

Quality filtering and adaptor trimming were performed using Trimmomatic version 0.39 (8). Default parameters were used for all software unless specified. Genomes were assembled using the Shovill pipeline version 1.1.0 (https://github.com/tseemann/shovill) and the St. Petersburg genome Assembler (SPAdes) version 3.15.5 (9). Annotations of the assembled genomes were carried out with Prokaryotic Genome Annotation Pipeline (PGAP) version 6.7 (10). The assemblies (Table 1) were checked for plasmid sequences with PlasmidFinder version 2.0.1 (11), with no plasmids found.

**TABLE 1:** Genomic features and GenBank accession numbers of the two *Roseibium* spp. isolates

Isolate	Genome size (bp)	Average coverage	No. of CDS^*a*^	No. of rRNAs	No. of tRNAs	G + C content (%)	No. of contigs	N_50_ (bp)	L_50_	Assembly accession no.	SRA accession no.	No. of reads	Average read length (bp)
SCP14	7,150,257	22.3	6,523	5	55	56.2	66	985,317	3	JBDZYH000000000	SRR28745651	12,463,498	151
SCP15	5,833,667	27.9	5,424	3	50	56.1	210	1,394,062	2	JBDZYI000000000	SRR28745650	10,920,523	151

^*a*^ CDS, coding DNA sequences.

Isolates were identified using the Microbial Genomes Atlas (12) web server using the NCBI-Prok function, with the closest similarity to *Labrenzia* sp. PHM005 (NZ_CP041191) and *Labrenzia* sp. VG12 (NZ_CP022529) based on average amino acid identity. Phylogenetic relatedness between each isolate and all publicly available *Roseibium* and *Labrenzia* reference genomes, as well as each other, was determined using the Orthologous Average Nucleotide Identity Tool, version 0.93.1 (13) and the Genome-to-Genome Distance Calculator, version 3.0 (14), to calculate the average nucleotide identity (ANI) and DNA-DNA hybridization (DDH) scores, respectively. Both genomes are <95% ANI and <70% DDH to each other and against all reference genomes, below the species threshold (15).

Both draft genomes, *Roseibium* sp. SCP14 and *Roseibium* sp. SCP15 (for Singapore coral probiotic), encoded genes for different isoforms of dimethylsulfoniopropionate (DMSP) lyases (*dddD* and *dddQ* for SCP14 and SCP15 respectively), which metabolize DMSP into the cloud nucleating agent dimethylsufide (16) and 3-hydroxypropionate, a precursor to the anti-herbivory compound acrylate (17). Further analyses with the Kyoto Encyclopedia of Genes and Genomes (18) database also revealed genes involved in denitrification in both genomes, which could function to maintain stable nitrogen levels within the coral holobiont (19).

## Data Availability

The SRA data were deposited to the National Center for Biotechnology Information, and the complete genome sequences were deposited to GenBank under the accession numbers listed in Table 1.
